# Protocol for binary food choice assays using *Drosophila melanogaster*

**DOI:** 10.1016/j.xpro.2022.101410

**Published:** 2022-05-18

**Authors:** Binod Aryal, Subash Dhakal, Bhanu Shrestha, Jiun Sang, Roshani Nhuchhen Pradhan, Youngseok Lee

**Affiliations:** 1 Department of Bio & Fermentation Convergence Technology, Kookmin University, Seoul 02707, Republic of Korea

**Keywords:** Genetics, Model Organisms, Neuroscience, Behavior

## Abstract

Food preference is a fundamental behavior for animals to choose nutritious foods while rejecting foods containing toxins. Here, we describe binary food choice assays using *Drosophila melanogaster*, which are straightforward approaches for the characterization of two-way choice tastants. We detail the preparation of flies and dye-containing food, followed by the binary-choice feeding assays and the determination of the preference index (PI). This protocol is simple, sensitive, and reproducible in qualitatively detecting attractive or aversive characteristics toward any two-way choice tastants.

For complete details on the use and execution of this protocol, please refer to Aryal et al. (2022).

## Highlights

• Protocol for qualitatively analyzing food preferences in *Drosophila*
• A simple, sensitive, and reproducible binary food choice assay
• Determination of preference index using dye-containing foods
• Appropriate for any behavioral taste analysis of fly models

## Abstract (editor highlights)

Publisher’s note: Undertaking any experimental protocol requires adherence to local institutional guidelines for laboratory safety and ethics.

## Graphical abstract

**Figure:**
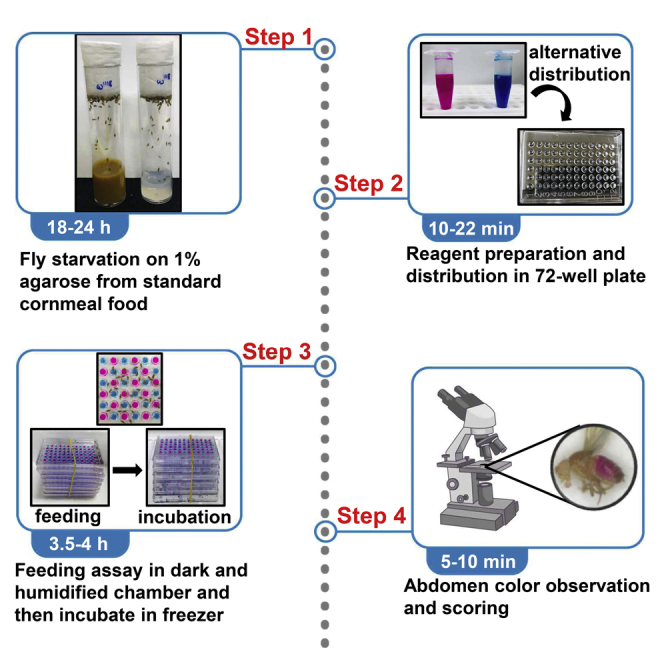

## Before you begin

Animals contain chemoreceptors for chemosensation, which helps them distinguish nutritious substances from toxic compounds. Chemosensation is further classified into two sensory components: smell and taste. Insects like fruit flies and mosquitoes have a capability of detecting a similar range of gustatory stimuli as those to humans, including bitter, sweet, sour, salt, and amino acids (Aryal et al., 2022; Liman et al., 2014). Many insects are endowed with gustatory organs distributed throughout the body, including the labellum, pharynx, legs, wing margins, and even the female genitalia (Chen and Dahanukar, 2020; Rimal and Lee, 2018; Vosshall and Stocker, 2007). However, labellum and legs are the major taste organs, which mediate a major role in contact chemosensation in insects. The full benefit of this behavioral assay is realized when performed in combination with an electrophysiological assay that measures tastant-induced action potentials in gustatory receptor neurons (tip recordings) (Aryal et al., 2022; Dhakal et al., 2021; Shrestha and Lee, 2021a).

For analysis of feeding behavior, different approaches have been employed for qualitative and quantitative analyses of food consumption. These include binary-choice feeding assays (Charroux and Royet, 2020; Tanimura et al., 1982; Zhang et al., 2013), the capillary feeder (CAFE) assay (Ja et al., 2007), Fly Liquid-Food Interaction Counter (FLIC) assay (Ro et al., 2014), radiolabel-based feeding (Deshpande et al., 2014; Thompson and Reeder, 1987), electronic-based feeding assays (Itskov et al., 2014; Ro et al., 2014), and Droso-X assay (Aryal and Lee, 2021; Sang et al., 2021). Binary-choice feeding assays are the most widely used behavioral approaches to monitor feeding, since they are simple to perform, safe, relatively inexpensive, can be used in a classroom setting and can be applied to large-scale genetic screening.

### Binary food choice assay

**Figure 1:**
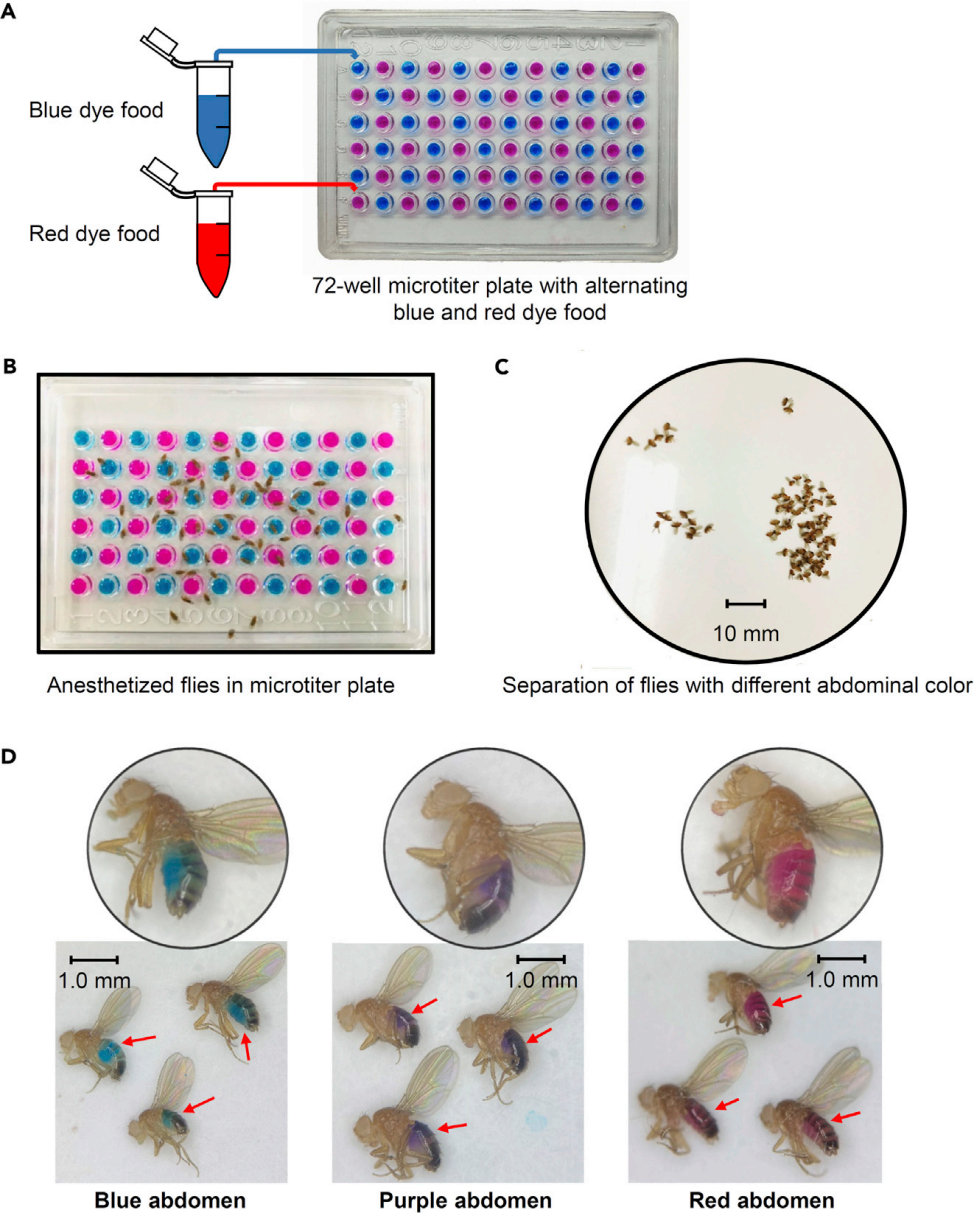
Binary food choice assay (A) Photograph of 72-well microtiter plate containing red dye and blue dye mixed food for a binary food choice assay. (B) Photograph of a microtiter plate containing anesthetized flies. (C) Photograph of ceramic plate in white background containing separated flies with different color abdomens. Scale bar indicates 10 mm. (D) Photographs of the flies with red abdomen, purple abdomen, and blue abdomen due to consumption of red dye, both dye, and blue dye containing food (indicated by red arrow). Scale bars indicate 1 mm.

Binary food choice assays can be easily used for identifying the feeding behavior of flies and are usually performed with 4–6-day old adult flies since they have anatomically and physiologically well-developed bodies (Meunier et al., 2003; Moon et al., 2006). The assays can be used to evaluate any two tastants by combining each tastants with either blue food coloring (brilliant blue FCF, 0.125 mg/mL) or red food coloring dyes (sulforhodamine B, 0.1 mg/mL) (Figure 1). The same sucrose concentration is used in both combinations to avoid bias, and the preference index (P.I) was calculated for each dye/tastant combination. We can also perform binary food choice assay without the addition of sugar in both the dye (Aryal et al*.*, 2022). Complete preference is indicated by P.I values of 1.0 and -1.0 while a P.I value of 0 indicates the absence of a bias between the two food choices (Aryal et al., 2022; Dhakal et al., 2021; Shrestha and Lee, 2021b; Shrestha et al., 2022).

Aversive compounds generally activate bitter-sensing or calcium-sensing gustatory receptor neurons (GRNs) (French et al., 2015; Lee et al., 2018). While attractive compounds activate sweet- and water-sensing GRNs. Furthermore, most aversive compounds inhibit sugar-sensing GRNs (Chu et al., 2014; French et al., 2015; Jeong et al., 2013; Lee et al., 2010, 2012; Meunier et al., 2003; Shrestha and Lee, 2021b). To overcome this sugar inhibition, a 5-fold higher concentration of sucrose is recommended for assays with aversive chemicals, based on our experience. Depending on the concentration of the aversive compounds used, flies can sense 5 mM sucrose like 1–3 mM sucrose because of the sugar inhibition. Indeed, this sugar inhibition has been explained by two mechanisms. First, stimulation of bitter-sensing GRNs activates GABAergic interneurons to inhibit sugar-sensing GRNs (Chu et al., 2014). Second, bitter compounds may bind to an odorant-binding protein in the endolymph to inhibit sugar-sensing gustatory receptors (Jeong et al., 2013). To test aversive compounds such as calcium, DEET, saponin, lobeline, umbelliferone, and strychnine (Lee et al., 2010, 2015, 2018; Poudel et al., 2015, 2017; Sang et al., 2019; Shrestha and Lee, 2021b), 1 mM vs 5 mM sucrose laced with different concentrations of aversive compounds were studied. Likewise, another approach can be employed by using 2 mM sucrose vs 2 mM sucrose with a chemical mixture if the aversion is relatively weak. Chemicals such as acetic acid and cucurbitacin B (cuc-B) can be tested using this combination when the aversiveness is relatively mild (Rimal et al., 2019, 2020). However, in some case binary food choice assay can be performed without sucrose if the testing chemicals have enough attractive cue (Aryal et al., 2022). After ingestion, the flies are sacrificed at −20°C and the examination of the abdomen is performed under a stereoscopic microscope. Regions showing red, purple, and blue colors are counted and the P.I is calculated as shown below.

The chemical sense of taste detection and discrimination is an important aspect to investigate the behavioral response of an animal to tastants. Since the development of binary food choice assays, these assays have been used to study various chemical sensations in *Drosophila*, and these studies produced both expected and unique results. Several previous studies have shown that binary food choice assays are useful for assessing the gustatory preferences of flies to a variety of attractive and aversive compounds. This assay is amenable to quantitative taste analysis by performing dose-response curves. When combined with loss-of-function mutations, binary expression systems and tools for activating and inhibiting neurons, binary food choice assays have proven to be powerful in unraveling the chemoreceptors (GRs, IRs and TRPs) and other signaling proteins that function in taste, and in identifying distinct classes of gustatory receptor neurons (GRNs) that function in attractive and aversive taste (Rimal and Lee, 2018). Recent studies have also shown that this assay can be combined with texture or with other sensory modality testing-methods (Jeong et al., 2016). These features are important, since food flavor may be dependent on the crosstalk among several different sensory modalities such as taste, olfaction, vision, and texture.

## Key resources table

**Table:** 

REAGENT or RESOURCE	SOURCE	IDENTIFIER
**Chemicals, peptides, and recombinant proteins**		

L-arginine	Sigma-Aldrich Co.	Cat. No. A5131
Caffeine	Sigma-Aldrich Co.	Cat. No. C8960
Cucurbitacin B (cuc-B)	Sigma-Aldrich Co.	Cat. No. 82226
Sucrose	Sigma-Aldrich Co.	Cat. No. S9378
Sulforhodamine B	Sigma-Aldrich Co.	Cat. No. 230162
Brilliant blue FCF	Wako Pure Chemical Industries Ltd.	Cat. No. 027-12842
Agarose, Sepro	GenDEPOT, USA	Cat. No. A0224-050
Saf-Instant Yeast Red	Societe Industrielle Lesaffre	Saf-Instant Yeast Red (https://saf-instant.com/en/professional/#gamme)

**Experimental models: Organisms/strains**		

*D. melanogaster*: *w*^*1118*^ (control), (4–6 days old adult, mixed sexes)	Bloomington Drosophila Stock Center	BL5905
*D. melanogaster*: *Gr93a*^*3*^, (4–6 days old adult, mixed sexes)	Bloomington Drosophila Stock Center (Lee et al., 2009)	BL27592
*D. melanogaster*: *Gr66a*^*ex83*^, (4–6 days old adult, mixed sexes)	Bloomington Drosophila Stock Center (Moon et al., 2006)	BL35528
*D. melanogaster*: *Gr33a*^*1*^, (4–6 days old adult, mixed sexes)	Bloomington Drosophila Stock Center (Moon et al., 2009)	BL31427

**Software and algorithms**		

Origin	OriginLab corporation	RRID: SCR_002815 (https://www.originlab.com/)
GraphPad Prism 8	GraphPad	RRID: SCR_002798 (https://www.graphpad.com/scientific-software/prism/)

**Other**		

72-well microtiter dishes	Thermo Fisher Scientific	Cat. No. 438733
Standard cornmeal food vial	Hansol Tech Co. Ltd., Republic of Korea	Cat. No. FFV-11010 (https://hansoltc.com/main/)
15 mL falcon tube	SPL Life Sciences Co. Ltd., Republic of Korea.	Cat. No. 50015
Standard 1.5 mL microcentrifuge tubes	SPL Life Sciences Co. Ltd., Republic of Korea.	Cat. No. 60015
0–2 μL, 2–20 μL, 20–200 μL, and 100–1,000 μL pipettes	Thermo Scientific, USA	Cat. No. 4700860N
Heat block (Thermo bath)	FINEPCR, Republic of Korea	Cat. No. ALB64
Microwave (800 W)	Magic chef	Cat. No. MEM-25S
Dissection microscope	Nikon, Japan	Cat. No. SMZ745
Deep freezer (−20°C)	Daewoo, Republic of Korea	Cat. No. FRS-530RE

## Materials and equipment

### Preparing stock solutions

• 1% agarose stock (wt/vol): Dissolve 1 gram of agarose in 100 mL double distilled water (ddH_2_O) by heating in a microwave. Allow it to cool down slightly. Can be stored at 20°C–25°C up to 1 week.
• 100 mM L-arginine stock (wt/vol): Dissolve 0.210 gram of L-arginine in 10 mL ddH_2_O. Can be stored at −20°C up to 3 months.
• 250 mM L-arginine stock (wt/vol): Dissolve 0.526 gram of L-arginine in 10 mL ddH_2_O. Can be stored at −20°C up to 3 months.
• 1,000 mM L-arginine stock (wt/vol): Dissolve 2.106 gram of L-arginine in 10 mL ddH_2_O. Can be stored at −20°C up to 3 months.
• 100 mM sucrose stock (wt/vol): Dissolve 3.423 gram of sucrose in 100 mL ddH_2_O. Can be stored at 4°C up to 1 month.
• 200 mM sucrose stock (wt/vol): Dissolve 6.846 gram of sucrose in 100 mL ddH_2_O. Can be stored at 4°C up to 1 month.
• 500 mM sucrose stock (wt/vol): Dissolve 17.115 gram of sucrose in 100 mL ddH_2_O. Can be stored at 4°C up to 1 month.
• 100 mM caffeine stock (wt/vol): Dissolve 1.942 gram of caffeine in 100 mL ddH_2_O. Can be stored at 4°C up to 1 month.

**CRITICAL:** Harmful if swallowed. Thus, the use of gloves and mask is recommended while handling this reagent.

• 10 mM cuc-B stock (wt/vol): Dissolve 0.0012 mg of cuc-B in 2 mL of 100% ethanol. Can be stored at 4°C up to 1 month.

**CRITICAL:** Fatal if swallowed. Thus, the use of gloves and mask is recommended while handling this reagent. Keep it sealed to prevent it from evaporating.

• 100× Blue dye (Brilliant blue FCF, 12.5 mg/mL (wt/vol)): Dissolve 125 mg of Brilliant Blue FCF in 10 mL ddH_2_O in a 15 mL Falcon tube. Prepare 1 mL aliquots and store them at 4°C. Can be stored at 20°C–25°C up to 1 month.
• 100× Red food dye (Sulforhodamine B, 10.0 mg/mL (wt/vol)): Dissolve 100 mg of sulforhodamine B in 10 mL ddH_2_O in a 15 mL Falcon tube. Prepare 1 mL aliquots and store them at 4°C. Can be store at 20°C–25°C up to 1 month.

## Step-by-step method details

### Fly strains

50–70 flies (4–6 days old; mixed sexes).

**CRITICAL:** Flies should be in healthy condition (without any physical damage) for bias-free results. Flies in each vial are transferred every 2 days in a humidified chamber.

### Preparation of mature fly for test

**Timing: ∼ 4–6 days**

This section describes how to maintain the flies before performing the assay.

1. Culture parent strains on a standard cornmeal food vial (25 mm diameter and 95 mm height) with addition of few grains of the yeast extract.
2. Collect newly enclosed flies for 0–2 days.
3. Transfer ∼50–70 collected flies to a new standard cornmeal food and aged for 4 more days at 25°C in a 12 h /12 h Light/Dark cycle, 50%–60% humidified incubator.
4. Transfer the fly to a new vial every 2 days. This prevents the adult flies from sticking to the fly food.

### Solution preparation

The following protocol describes the preparation of 1 mL solutions containing 1 mM or 5 mM or without sucrose with different color dyes. Prepare these solutions just before distributing them on a microtiter plate. Other attractive tastants (e.g., sugars) can also be tested using this assay. To test the tastants (e.g., 25 mM arginine and 100 mM arginine) without sugar, add 1 mM arginine to one colored dye and 25 mM (follow steps 5 and 6) or 100 mM tastants (follow steps 5 and 7) to another colored dye. Similarly, to test the effects of a bitter tastant (e.g., 10 mM caffeine), add the aversive compound to the 5 mM sucrose, which is tested against the 1 mM sucrose alone (follow steps 8 and 9). One color dye should be added to the 1 mM sucrose and the other dye to the 5 mM sucrose. We will also explain below how to prepare 2 mM sucrose alone versus 2 mM sucrose combined with cuc-B (follow steps 10 and 11) or 1 mM L-arginine versus 25 or 100 mM L-arginine. In the initial experiments, set up controls in which the dyes colors are switched to control for biases caused by the dyes. After the ‘follow ∼ to ∼’ process mentioned above, go to step 12. If dye bias happens, please refer to ‘problem 1’ for potential solutions.

### Preparation of 1 mM of L-arginine solution

**Timing: ∼ 5–10 min**

**Table:** 

Reagent	Final concentration	Amount
L-arginine (100 mM)	1 mM	10 μL
Red or blue dye (100×)	1×	10 μL
1.0% agarose	0.89%	890 μL
ddH_2_O	n/a	90 μL
**Total**	**n/a**	1,000 μL

5. Mix the components completely by vortexing and immediately dispense in microtiter plates as described below in step 12.

**Pause point:** Place in a 57°C heat block for ≤30 min if immediate dispersion cannot be performed.

***Note:*** If the final volume before addition of agarose to any of the dye side is higher then add d/w to the lower side to make an equal volume to both the side.

### Preparation of 25 mM L-arginine solution

**Timing: ∼ 5–10 min**

**Table:** 

Reagent	Final concentration	Amount
L-arginine (250 mM)	25 mM	100 μL
Red or blue dye (100×)	1×	10 μL
1.0% agarose	0.89%	890 μL
ddH_2_O	n/a	0 μL
**Total**	**n/a**	1,000 μL

6. Mix the components completely by vortexing and immediately dispense in microtiter plates as described in step 12.

**Pause point:** Place in a 57°C heat block for ≤30 min if immediate dispersion cannot be performed.

***Note:*** If the final volume before addition of agarose to any of the dye side is higher then add d/w to make an equal volume to both the side.

### Preparation of 100 mM L-arginine solution

**Timing: ∼ 5–10 min**

**Table:** 

Reagent	Final concentration	Amount
L-arginine (1,000 mM)	100 mM	100 μL
Red or blue dye (100×)	1×	10 μL
1.0% agarose	0.89%	890 μL
ddH_2_O	n/a	0 μL
**Total**	**n/a**	1,000 μL

7. Mix the components completely by vortexing and immediately dispense in microtiter plates as described in step 12.

**Pause point:** Place in a 57°C heat block for ≤30 min if immediate dispersion cannot be performed.

***Note:*** If the final volume before addition of agarose to any of the dye side is higher then add d/w to make an equal volume to both the side.

### Preparation of 1 mM sucrose solution

**Timing: ∼ 5–10 min**

**Table:** 

Reagent	Final concentration	Amount
Sucrose (100 mM)	1 mM	10 μL
Red or blue dye (100×)	1×	10 μL
1.0% agarose	0.88%	880 μL
ddH_2_O	n/a	100 μL
**Total**	**n/a**	1,000 μL

8. Mix the components completely by vortexing and immediately dispense in microtiter plates as described in step 12.

**Pause point:** Place in a 57°C heat block for ≤30 min if immediate dispersion cannot be performed.

### Preparation of 5 mM sucrose solution

**Timing: ∼ 5–10 min**

**Table:** 

Reagent	Final concentration	Amount
Sucrose (500 mM)	5 mM	10 μL
Red or blue dye (100×)	1×	10 μL
1.0% agarose	0.88%	880 μL
Caffeine (100 mM)	10 mM	100 μL
**Total**	**n/a**	1,000 μL

***Optional:*** Add an aversive tastant (for adding 10 mM caffeine from 100 mM stock solution, add 100 μL of caffeine solution).

***Note:*** If solution is more than 200 μL then concentration of agarose should be increased to balance the rigidity.

9. Mix the components completely by vortexing and immediately dispense in microtiter plates as described in step 12.

**Pause point:** Place in a 57°C heat block for ≤30 min if immediate dispersion cannot be performed.

### Preparation of 2 mM sucrose alone solution

**Timing: ∼ 5–10 min**

**Table:** 

Reagent	Final concentration	Amount
Sucrose (200 mM)	2 mM	10 μL
Red or blue dye (100×)	1×	10 μL
1.0% agarose	0.979%	979 μL
ddH_2_O	n/a	1 μL
**Total**	**n/a**	1,000 μL

10. Mix the components completely by vortexing and immediately dispense in microtiter plates as described in step 12.

**Pause point:** Place in a 57°C heat block for ≤30 min if immediate dispersion cannot be performed.

### Preparation of 2 mM sucrose with tastant solution

**Timing: ∼ 5–10 min**

**Table:** 

Reagent	Final concentration	Amount
Sucrose (200 mM)	2 mM	10 μL
Red or blue dye (100×)	1×	10 μL
1.0% agarose	0.979%	979 μL
cuc-B (10 mM)	0.01 mM	1 μL
**Total**	**n/a**	1,000 μL

***Optional:*** Add an aversive tastant (for adding 0.01 mM cuc-B from 10 mM stock solution, add 1 μL of test solution).

**CRITICAL:** Some aversive compounds are not soluble in water or have very low solubility in water. You may use ethanol as a solvent and insert the same amount of ethanol in both sides. For example, we used absolute ethanol to dissolve cuc-B (Rimal et al., 2020) and 50% ethanol for umbelliferone to make stock solution (Poudel et al., 2015).

11. Mix the components completely by vortexing and immediately dispense in microtiter plates as described in step 12.

**Pause point:** Place in a 57°C heat block for ≤30 min if immediate dispersion cannot be performed.

**CRITICAL:** To ensure that the dyes have minimal effects, the exact concentrations of the red and blue food need to be re-established empirically for each batch of food dye. See: Dye dose-response curves.

**CRITICAL:** If a volatile chemical such as DEET is to be tested, add the chemical immediately before dispensing the 5 mM sucrose solution on the microtiter plates to avoid evaporation. Beginners would better be trained to dispense the solutions to make 1 plate in 2 min.

### Preparing 72-well microtiter dishes for binary-choice feeding assays

**Timing: dispensing, 1–2 min per dish; use between 10 min to 2 h after dispensing**

This section describes dispensing process of dye containing food to 72-well microtiter dishes.

12. Distribute 11 μL of the two types of tastants in an alternative fashion into 72 well microtiter dishes (Figure 1A). For the dispensing of the solution, regular 0–20 μL pipette can be used.

**CRITICAL:** This step should be done quickly. The distribution should be finished within 30 min since prolonged exposure to air may cause dehydration, therefore changing the food texture.

**CRITICAL:** The microtiter dishes should be closed with the microplate lid immediately after the dye/tastants distribution. This will prevent any contamination and evaporation.

13. Start next step in 30 min.

### Preparing flies for binary-choice feeding assays

**Timing: ∼ 18–24 h**

This section describes fly preparation for maintaining flies before the binary food choice assays begin.

14. To induce feeding during the food choice assay, starve the flies by incubating them in a vial containing 1% agarose only for 18–24 h (humidity: 50%–60%, temperature: 25°C, Light/Dark: 12/12 h).

***Note:*** The flies gross motor activities must not be significantly reduced after starvation, since this may decrease exploration and result in inaccurate or non-reproducible results. It should be noted that starvation can alter feeding motivation independent of the sense of taste, due to effects on the metabolic state, changes in the expression of hormones and proprioceptive feedback from the gut and crop. Depending on the internal nutritional state, starvation may affect taste perception. Furthermore, starvation can reduce aversion to bitter compounds and reduces attraction to sugars (LeDue et al., 2016). Regarding the problem raised by the no color observation of the abdomen, please refer to ‘problems 2 and 3’ for potential solutions.

**CRITICAL:** To avoid subjectivity, code the vials at this point so that the results can be scored in a blind manner.

### Conducting and analyzing binary-choice feeding assays

**Timing: ∼ 5–6 h**

This section describes the process of transferring starved flies to microtiter dishes, incubation process for allowing the feeding, sacrificing the fed flies and calculating the preference index based on color of abdomens.

**CRITICAL:** Perform the assays between ZT_0_– ZT_5_ because circadian rhythm can affect the amount of food intake.

**CRITICAL:** The binary-choice feeding assays should be performed in a controlled chamber with temperature of 25°C and 60% humidity.

15. Place the vials containing the starved flies in an ice-water mixture for 2–5 min to stop the animals motor activity.
16. Gently transfer ∼50–70 flies into the middle of each microtiter plate, and immediately cover the plates (Figure 1B).
17. Put the plates into a dark, humidified box, and allow the flies to consume the food for 90 min in the dark. Dark environment is to reduce the influence of dye color to visual cues.

***Note:*** 1.5 h is typically the optimal feeding period. The decrease in the PI after 2.5 h may be a consequence of food adaptation.

18. After 90 min, transfer the microtiter plates to a −20°C freezer for 90–120 min. The plates should be inverted to allow the sacrificed flies to fall onto the lids.
19. Score the number of flies with red (NR), blue (NB) or purple abdomens (NP) using a stereomicroscope (Figure 1C).

***Note:*** Ideally, the number of flies that can be scored is ≥80%, to minimize variability resulting from scoring low numbers of flies. If sexual dimorphic effects are doubted, each gender should be retested separately.

**Figure 2:**
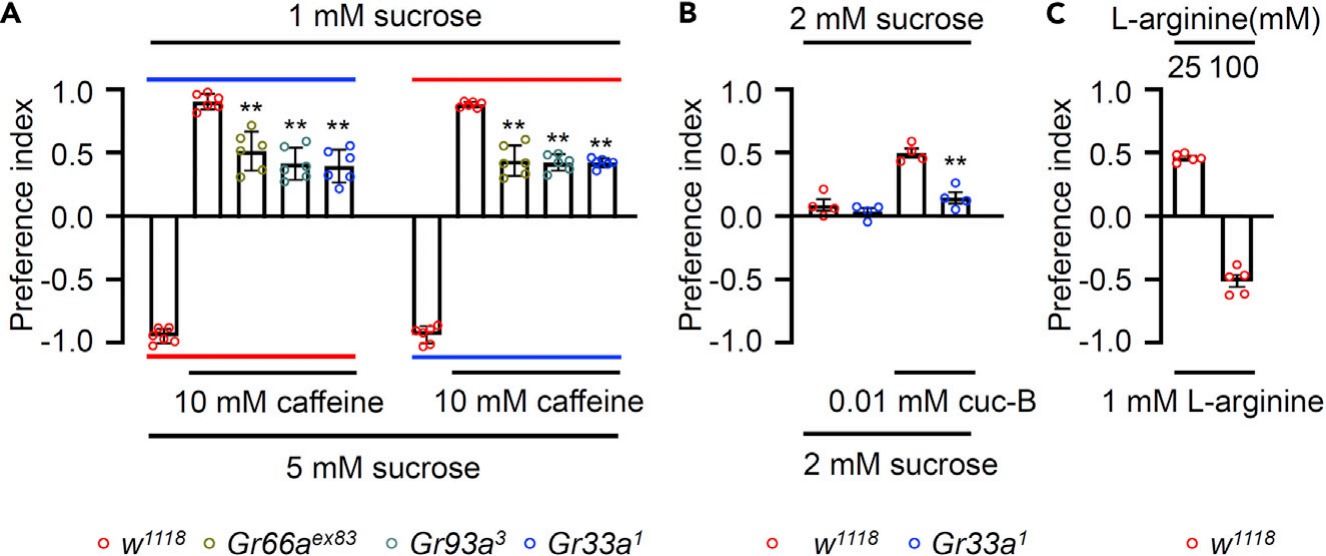
Three different set-ups for binary food choice assays (A) Binary food choice assays of 10 mM caffeine in 5 mM sucrose vs 1 mM sucrose condition with *w*^*1118*^ (control), *Gr33a*^*1*^*, Gr66a*^*ex83*^, and *Gr93a*^*3*^ mutants. Two color dyes (red and blue) mixed with alternative food choices were switched to exclude possible preferences to dye. Blue and red lines indicate the dye color mixed with the food and flies’ preference to that dye containing food (n = 6). (B) Binary food choice assays of *w*^*1118*^ (control) and *Gr33a*^*1*^ at 0.01 mM cuc-B (n= 4). (C) Binary food choice assays of *w*^*1118*^ (control) flies performed with 1 mM L-arginine versus 25 mM or 100 mM L-arginine concentration (n= 5). All error bars represent SEMs. Single-factor ANOVA coupled with Scheffe’s post hoc analysis was conducted to compare multiple sets of data. Asterisks indicate statistical significance compared with the control and mutant, respectively (∗∗p < 0.01).

20. Determine the preference index (PI) according to the formula below. The noncolored flies are not included when calculating the PI (Figures 2A–2C).

$$\text{PI}=\left({\text{N}}_{\text{B}}-{\text{N}}_{\text{R}}\right)/\left({\text{N}}_{\text{R}}{+\text{N}}_{\text{B}}{+\text{N}}_{\text{P}}\right)\text{ or }\left({\text{N}}_{\text{R}}-{\text{N}}_{\text{B}}\right)/\left({\text{N}}_{\text{R}}{+\text{N}}_{\text{B}}{+\text{N}}_{\text{P}}\right)$$

### Dye dose-response curves

This section describes how to maintain dye concentration so that the dyes do not affect the result of PI.

**CRITICAL:** To establish the optimal concentrations of red and blue dyes, it is critical to identify dye concentrations that are not aversive, as this would influence the outcome of the binary-choice feeding assays. In addition, sufficient dye must be used so that it is possible to detect red, blue, and purple colors in the abdomens following the 90-minute feeding interval. We typically use 1× (0.125 mg/mL of blue dye and 0.1 mg/mL of red dye) concentrations of the dyes.

***Note:*** It is possible to use up to 10-fold less dye of one color than the other, and still assess both blue and red abdomens.

***Note:*** Because high dye concentrations make it easier to score the color of the abdomens, the goal is to establish the highest concentrations of red and blue dyes (up to 1×) that are not aversive or attractive.

21. Because 1× (0.125 mg/mL of blue dye and 0.1 mg/mL of red dye) of each dye is usually acceptable, mix 1× red dye and 1× blue each with 1 mM or 2 mM or 5 mM sucrose or without sucrose, and distribute in a zigzag pattern into 72 well microtiter dishes (Figures 1A and 1B). Also, to avoid any bias raised due to dye effect, dye switching test can be performed in the same paradigm. This switching of tastant/dye combinations does not affect food preference (Figures 2A and 2B).

***Note:*** We recommend the number of trials of dye switching should be ≥ 3.

22. Determine the PI and the percentage of animals that can be scored. This is based on the visibility of the dye in abdomen (Figure 1D). If food consumption is low, then it is difficult to detect the color in the abdomen.
23. Interpretations.
  a. **Neither dye is aversive:** The ideal results would be a PI=0 and ≥80% of flies that can be scored based upon the visibility of colored abdomen. If this is the case, no additional tests are necessary, and you can use 1× (0.125 mg/mL of blue dye and 0.1 mg/mL of red dye) concentrations of each dye.
  b. **One dye is aversive:** If the PI is outside of the range of 0 but the percentage of flies that can be scored is ≥80% based upon the visibility of colored abdomen, then just one dye is excessively aversive.
  c. **Both dyes are aversive:** If the PI is outside of the range of 0, and the percentage of flies that can be scored is <80% based upon the visibility of a colored abdomen, then both dyes are excessively aversive. Regarding the problems of the dose-dye effect, please refer to ‘problems 4, 5, 6’ for potential solutions.

## Expected outcomes

Following the protocol, we performed the binary-choice feeding assay of test chemicals in 1 mM vs 5 mM sugar paradigm and 2 mM vs 2 mM sugar paradigm taking both control and mutant flies. We also tested binary food choice assays without sucrose for L-arginine at low (25 mM) and high (100 mM) vs 1 mM L-arginine. We applied chemical plus sucrose food in one dye and only sucrose in the other dye. We used aversive compound like cuc-B for 2 mM vs 2 mM sucrose condition and caffeine for 1 mM vs 5 mM sucrose condition. Since, both compounds are bitter in taste, we expected flies to avoid these bitter compounds containing foods as an aversive taste cue. As expected, both the compounds (cuc-B and caffeine) were avoided by the control animals while mutants exhibited deficiency in avoiding these compounds (Figures 2A and 2B) as preference index of mutant was significantly reduced compared to the control. Furthermore, we found that control flies attract 25 mM L-arginine, but avoid 100 mM L-arginine (Figure 2C). Three different combinations can allow us to evaluate preference of tastants.

## Limitations

The chemical sense of taste detection and discrimination is an important aspect to investigate the behavioral response of an animal to tastants. Although the binary food choice assay plays a suitable role in identifying genes encoding receptors and other proteins, the assays do not allow discrimination between a requirement for genes in the afferent neurons—the gustatory receptor neurons (GRNs), and a requirement for genes in the central brain. The neurological signal which is generated and passed to the brain also could not be analyzed. Furthermore, the role of single specialized sensory hairs (sensilla) could not be predicted based upon the behavioral assay which can limit the physiological analysis. For these analyses, a combination of a binary food choice assay and an electrophysiological assay is recommended.

An inherent complication in analyzing the behavioral effects of aversive tastants using binary food choice assays is that most bitter chemicals along with acid and high concentration of salt can suppress sugar-activated GRNs (Chen et al., 2019; Jeong et al., 2013). Therefore, to equilibrate the sweetness between two sides, we compare the behavioral responses of a bitter compound added to a higher sugar concentration (5 mM sucrose), relative to a lower concentration of sugar only (1 mM sucrose). We use these two different sugar concentrations since we found that mutations which eliminate the responses of bitter-activated GRNs to intermediate concentrations of bitter compounds such as caffeine, lobeline, umbelliferone, strychnine, saponin, DEET and others, usually result in balanced selection of 1 mM versus 5 mM sucrose (PI=∼0 (Sang et al., 2019; Shrestha and Lee, 2021b).

Further, we recommend for investigators to establish the optimal concentrations of red and blue food dyes that have minimal effects on the assay prior to conducting all the trials for a given project, thereby preventing any bias for one side or the other based on the color of the food dye. To avoid any dye-based influence, a dye-switching test is recommended with the same paradigm and equal amounts of trials.

In rare instances wherein a binary food choice assay cannot be performed due to poor food consumption, it may be possible to employ the proboscis extension response (PER) assay, which involves touching a positive tastant, such as sucrose, to taste sensilla, and observing the innate extension of the proboscis (Dhakal et al., 2021; Rimal and Lee, 2019). However, this assay must be performed on single flies, as it is more time-consuming than binary food choice assays and requires more training to get consistent results.

One of the important limitations of binary food choice assay is that the observed feeding defects in mutants could be due to multiple taste organs such as labellum, legs, wing margin, or pharynx. Therefore, further genetic dissection such as electrophysiological assay, PER assay, and organs ablation experiments are required for attributing behavioral effects to specific taste organs.

## Troubleshooting

### Problem 1

Dye bias. Possible reason**:** The concentration of either the Brilliant Blue FCF or the sulforhodamine B, might be too high, resulting in an aversive response. This might occur due to differences among food dyes derived from different batches (steps 5–11).

### Potential solution

Optimize dye concentration by performing a dye dose response curve (see dye dose-response curves section).

### Problem 2

Reduced motility of flies after starvation. Possible reason: Fly strain may have mutations that reduce glycogen stores or glycogen mobilization (step 14).

### Potential solution

Decrease starvation time. If this results in low food consumption, increase feeding from 90 to 120 min. Avoid feeding >120 min, as the flies may adapt to some bitter tastants during prolonged exposure.

### Problem 3

Inconsistent results arising from different mutant alleles. Possible reason: Many behavioral assays including binary food choice assays, can be sensitive to the genetic background (step 14).

### Potential solution

Outcross the mutant flies to the “wild-type” control for at least five generations.

### Problem 4

<80% of the flies have colored abdomens and can be scored. Possible reason: Detecting abdominal colors can be difficult if:

• the animals eat an insufficient amount of food (step 23).
• the cuticle is too dark to score the food color inside the midgut (step 23).
• the animals eat a spectrum of blue and red food mixture, making it difficult to simply assign either blue or red to the animals (step 23).

### Potential solution

• Increase starvation time from 18 to 24 h up to 36 h.
• Increase feeding time from 90 min to 120 min.
• Dissect the abdomen to check the color of midgut and crop.

### Problem 5

Dye dose-response: PI is outside of the 0.0 range, but the % of flies that can be scored is ≥80%. Possible reason: One dye may be excessively aversive (step 23).

### Potential solution

Repeat the experiments with 1.0× of the preferred dye, and a series of lower levels of the other dye (0.8×, 0.6×, 0.4×, 0.2× and 0.1×).

### Problem 6

Dye dose-response: PI is outside of the 0.0 range, and the % of flies that can be scored is <80%. Possible reason: Both dyes are excessively aversive (step 23).

### Potential solution

Perform a titration using a range of red and blue dye concentrations from 0.8× to 0.1× (25 combinations).

## Resource availability

### Lead contact

Further information and requests for resources and reagents should be directed to and will be fulfilled by the lead contact, Youngseok Lee (ylee@kookmin.ac.kr).

### Materials availability

This study did not generate new unique reagents or chemicals.

## Data Availability

The code supporting the current study is available from the corresponding author on request.
